# Mutant p53 accumulates in cycling and proliferating cells in the normal tissues of p53 R172H mutant mice

**DOI:** 10.18632/oncotarget.4956

**Published:** 2015-07-22

**Authors:** Amanda M. Goh, Yuezhen Xue, Marc Leushacke, Ling Li, Julin S. Wong, Poh Cheang Chiam, Siti Aishah Binte Rahmat, Michael B. Mann, Karen M. Mann, Nick Barker, Guillermina Lozano, Tamara Terzian, David P. Lane

**Affiliations:** ^1^ p53 Laboratory, A*STAR, Singapore; ^2^ Institute of Medical Biology, A*STAR, Singapore; ^3^ Institute of Molecular and Cell Biology, A*STAR, Singapore; ^4^ Department of Genetics, The University of Texas MD Anderson Cancer Center, Houston, TX, USA; ^5^ Cancer Research Program, Houston Methodist Research Institute, Houston, TX, USA

**Keywords:** mutant p53, p53 R172H mouse model, small intestine, proliferation, Mdm2 inhibitor

## Abstract

The tumour suppressor p53 is regulated primarily at the protein level. In normal tissues its levels are maintained at a very low level by the action of specific E3 ligases and the ubiquitin proteosome pathway. The mutant p53 protein contributes to transformation, metastasis and drug resistance. High levels of mutant p53 can be found in tumours and the accumulation of mutant p53 has previously been reported in pathologically normal cells in human skin. We show for the first time that similarly elevated levels of mutant p53 can be detected in apparently normal cells in a mutant p53 knock-in mouse model. In fact, in the small intestine, mutant p53 spontaneously accumulates in a manner dependent on gene dosage and cell type. Mutant p53 protein is regulated similarly to wild type p53, which can accumulate rapidly after induction by ionising radiation or Mdm2 inhibitors, however, the clearance of mutant p53 protein is much slower than wild type p53. The accumulation of the protein in the murine small intestine is limited to the cycling, crypt base columnar cells and proliferative zone and is lost as the cells differentiate and exit the cell cycle. Loss of Mdm2 results in even higher levels of p53 expression but p53 is still restricted to proliferating cells in the small intestine. Therefore, the small intestine of these p53 mutant mice is an experimental system in which we can dissect the molecular pathways leading to p53 accumulation, which has important implications for cancer prevention and therapy.

## INTRODUCTION

The p53 tumour suppressor protein is a transcription factor that activates genes responsible for acute radiation-induced death, cell cycle checkpoint function, induction of apoptosis and senescence and tumour suppression. Its inactivation is considered a key event in human carcinogenesis. As a “guardian of the genome” [1], in normal tissues p53 protein is rapidly activated by different cellular stress pathways, such as DNA damage, hypoxia, spindle damage or oncogenic stimuli. p53 is stabilized and post-translationally modified to serve its transcriptional activation function. The target genes of p53 include Mdm2, which targets p53 for proteasomal degradation, enabling an important negative feedback loop that restores the low basal levels of p53 after activation [2-6].

The p53 protein is essential for the regulation of cell proliferation, and mutant p53 over-expression is usually seen in malignant tumours. Here the missense mutant protein accumulates to high levels and extensive immunohistochemical studies have shown that this accumulation is highly variable and that mutation alone is not sufficient to drive accumulation in all cells in the tumour leading to complex and variable staining patterns [7]. Mutation of the p53 gene results in loss of its tumour suppressive transcriptional activation properties and gain of novel oncogenic functions that are dependent on high level expression [8]. Germline mutations in p53 cause Li-Fraumeni Syndrome, which is characterised by the early onset of cancer in a wide variety of possible tissue types [9,10]. Genetic and xenograft data from mouse models have shown that the restoration of wild type p53 activity is a potential anticancer strategy [11-16].

Small intestinal epithelium has a remarkable rate of self-renewal which provides a daily readout of proliferative activity [17]. The epithelium of the murine small intestine renews every 5 days [18,19]. Each small intestinal crypt contains approximately six long-lived stem cells (crypt base stem cell, CBC) and these cells divide every day [20]. Their daughter cells consist of the transit-amplifying (TA) crypt compartment and these cells divide every 12-16 hours. They perform up to five rounds of cell division while migrating upwards [21]. The Paneth cells located between CBC cells reside at the crypt base for 3-6 weeks [22]. When TA cells reach the crypt-villus junction, they rapidly differentiate as villous epithelium. We have made use of the murine small intestine system to study mutant p53 expression in order to understand p53 expression in various cell populations of morphologically normal tissues and its association with potential function in initiation of preneoplasia/neoplasia.

We generated mice carrying a missense p53 R172H mutation which corresponds to the p53 R175H hot spot mutation in human tumours, and is associated with Li-Fraumeni Syndrome [23, 24]. This particular hot spot mutation in the p53 DNA-binding domain results in a protein that is transcriptionally inactive and has both dominant negative and gain-of-function phenotypes [8]. It is generally considered that p53 is mainly regulated at the protein rather than the transcriptional level, and mutant p53 protein is stable in human cancers but unstable in normal tissues [25]. In previous studies in mouse [25] and zebrafish [26] models where the animal's wild type p53 genes had been eliminated and replaced with mutant p53. It was reported that the mutant p53 proteins did not accumulate in normal tissues but reached very high levels of expression in the tumours that arose in these animals. In both the mouse and Zebrafish models the inactivation of the Mdm2 gene in a homozygous mutant p53 background results in the high level expression of the mutant p53 in many normal tissues [25, 26]. In this study, we show that mutant p53 protein is detectably expressed in a specific cell population of morphologically normal small intestine and also in other tissues in both absence and presence of p53-activating signals and even in the presence of active Mdm2. This level of expression is much higher than that seen for the wild type protein in unstressed tissues. We show using the Mdm2 inhibitor Nutlin *in vivo* that the mutant p53 levels are further increased by pharmacological inhibition of Mdm2 and are also induced by DNA damage. Importantly this allowed us to explore the possibility that down-regulation of wild type and mutant p53 protein in differentiated small intestinal epithelium occurs at the transcriptional level and to detect gene dosage effects on protein expression.

## RESULTS

### Heterogeneous expression of p53 R172H protein in morphologically normal adult mouse tissues

**Figure 1:**
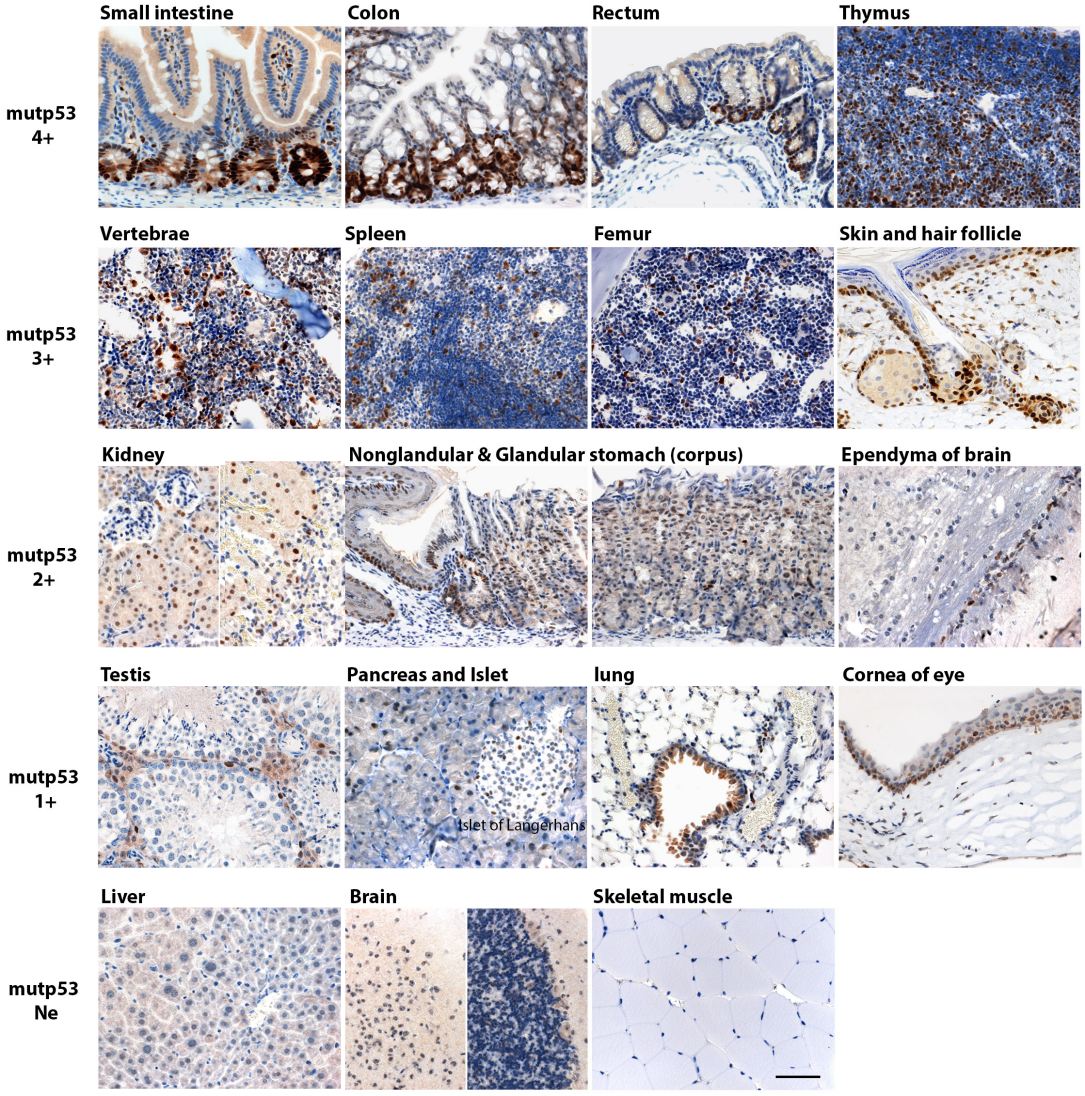
Mutp53 accumulation in morphologically normal multiple tissues in p53 R172H mice Adult p53 R172H mice were sacrificed in 2 mo and multiple tissues were harvested and embedded in paraffin block for immunostaining with p53 antibody. Tissues are divided into four groups according to p53 expression levels and positive cell fraction: mutp53 4+: small intestine, colon, rectum and thymus; mutp53 3+: bone marrow of vertebrae and femur, spleen, growing skin and hair follicle; mutp53 2+: kidney, nonglandular & glandular stomach and ependyma of brain; mutp53 1+: testis, pancreas & islet of Langerhan, lung, and cornea of eye. Mutp53 Ne (Negative): liver, brain (except ependyma) and skeletal muscle. Scale bar: 50 μm.

R172H mutant p53 protein (mutp53) levels may be elevated in preneoplastic cells, therefore we examined morphologically normal tissues in the *p53^R172H/R172H^* mice to study the expression of mutp53. We found that in the majority of apparently normal adult mouse tissues, there is a heterogeneous expression of mutp53 and we were able to divide mutp53 accumulation in mouse organs into four groups according to staining intensity and positive cell fraction in the population (Figure 1). Mutp53 4+: small intestine, colon, rectum and thymus; mutp53 3+: bone marrow of vertebrae and femur, spleen, growing skin and hair follicle, mutp53 2+: kidney, nonglandular & glandular stomach and ependyma of brain; mutp53 1+: testis, pancreas & islet of Langerhan, lung and cornea. There is no detectable immunostaining of mutp53 in liver, brain (except ependyma) and skeletal muscle.

In *p53^R172H/R172H^* mice, mutp53 accumulation was confined to the crypts of the small intestine. In colon and rectum, mutp53+ was accumulated in lower 2/3 of crypts. Mutp53 accumulation in thymus was detected both in cortex and medulla and was more pronounced in the medullary compartment. In spleen, mutp53+ cells distribute both in red pulp and white pulp, more mutp53+ cell populations are located in the red pulp. In bone marrow of vertebrae and femur, scattered mutp53 immuno-positive cell populations were found amongst the hematopoietic cells. Mutp53 accumulation is observed in growing skin and anagen hair follicles. In kidney, mutp53 expression is only found in the proximal convoluted tubules located in renal cortex while mutp53 was undetectable in glomeruli and medulla. Mutp53 accumulated in basal layers of nonglandular stomach and scattered expression is seen in the upper part of corpus region of stomach. Mutp53 is only expressed in the spermatogonium of testis, and the ependyma of brain. Mutp53 expression is not prominent in pancreas and lung. Mutp53 is immunonegative in liver, brain (except ependyma) and skeletal muscle. There was very weak or non-identifiable p53 staining in p53 wild type mouse tissues, and as expected there was no p53 immunopositive staining found in p53 knockout mice. Intriguingly, in *p53^R172H/KO^* mice, which bear a single mutant p53 allele, p53 R172H protein was found at low levels in all the tissues in which we detected mutp53 staining in the *p53^R172H/R172H^* mice. Therefore, p53 protein levels in tissue of p53 R172H mice are dependent on gene dosage (Suppl Figure 1).

### p53 R172H protein accumulates in intestinal crypts in a manner dependent on cell type and gene dosage

**Figure 2:**
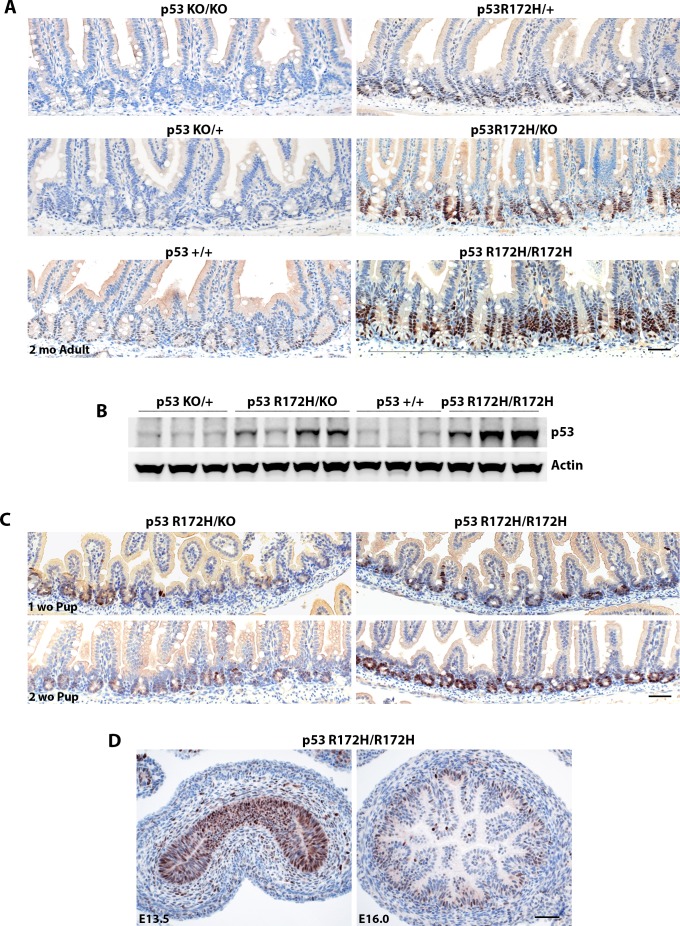
p53 R172H protein accumulation is dosage dependent and cell type specific **A.** Mutp53 expression patterns in different p53 genotypes as indicated. **B.** Western blot detection of total p53 protein levels in various p53 genotypes. **C.** & **D.** Accumulation of p53 R172H protein is detectable in small intestine of postnatal pups and in gut tube as well as in nascent crypts of embryos (E13.5 & E16). Scale bar: 50 μm.

**Figure 3:**
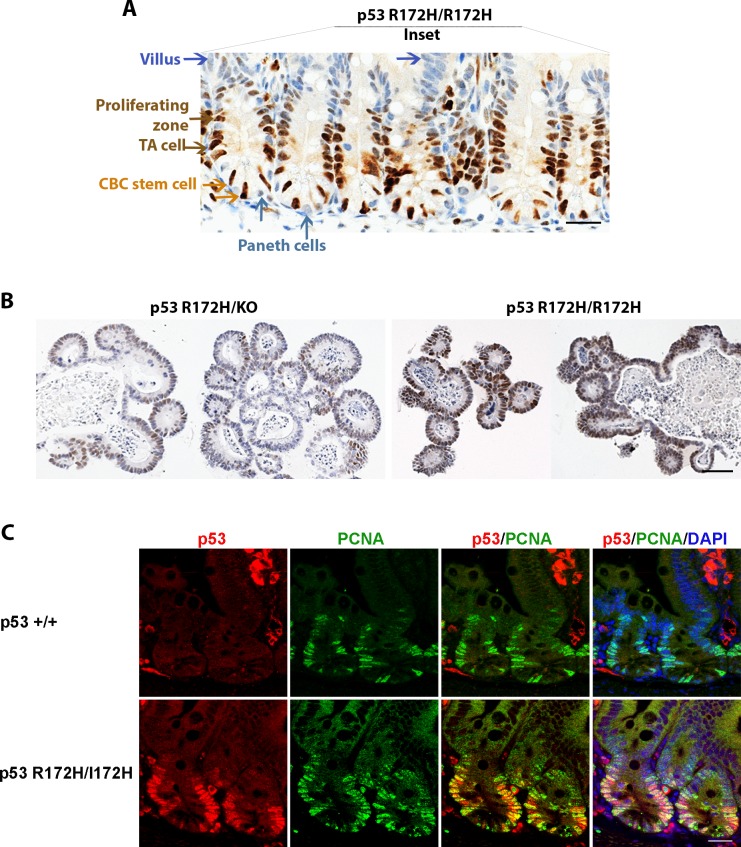
p53 R172H expression in cycling and proliferative cells of small intestinal crypts **A.** mutp53 expressed in cycling crypt base columnar (CBC) stem cells and proliferating transit-amplifying (TA) cells but is absent in differentiated Paneth cells and villous epithelia. Orange arrow: CBC stem cell, Brown arrow: proliferating TA cells. Cyanic arrow: Paneth cells, Blue arrow: villi. **B.** *ex vivo* cultures of intestinal crypts from p53 R172H mice mimic the gene dosage phenotype observed *in vivo*. **C.** Co-labelling of p53 and proliferative marker PCNA in p53 wild type and p53 R172H mouse small intestine. Scale bar: 20 μm.

The pattern of p53 immunopositivity in the small intestine (Figure 2A, 3A, Suppl Figure 2 and Cover page) was particularly interesting. No p53 protein was detected in p53-null (*p53^−/−^*) and very weak or low levels of p53 protein in p53 wild type (*p53^KO/+^ and p53^+/+^*) mouse small intestines. In *p53^R172H/+^* and *p53^R172H/KO^* mice, which bear only a single mutant p53 allele, p53 R172H protein was found at lower levels in a majority of the crypts (still higher than p53 wild type mice) with a few occasional strong immunopositive foci in crypts of *p53^R172H/KO^ mice*. In *p53^R172H/R172H^* mice, p53 R172H protein levels were elevated in all the crypts of the small intestine, the specificity of strong p53 staining by IHC in the small intestine of *p53^R172H/R172H^* mice was also verified by our home-made p53 polyclonal rabbit antibody which reacts strongly with p53 and has no background staining in p53-null mice using immunofluorescent staining (Suppl. Figure 2). Therefore, p53 protein levels in the crypts of the small intestine are dependent on genotype and gene dosage, which was further validated by Western blot when we extracted protein from duodenum and jejunum of small intestine in *p53 wild type (p53^+/+^), p53^KO/+^, p53^R172H/KO^, and p53^R172H/R172H^* mice to compare total mutp53 protein expression levels between one allele or two alleles of the p53 R172H mutant gene (Figure 2B). We could detect elevated and dosage dependent mutp53 protein expression in *p53^R172H/KO^ and p53^R172H/R172H^* mouse small intestine, while only weak bands were detected in *p53 wild type* mice.

We could detect increased p53 R172H protein in the intestinal crypts of *p53^R172H^* pups as early as one week after birth. The effect of gene dosage on p53 protein levels was very clear in pups at just two weeks of age (Figure 2C). In *p53^R172H/R172H^* pups, p53 R172H protein levels were high in every crypt and remained low but detectable in their p53R172/KO littermates. Strong mutp53 staining is also observed in pseudostratified epithelium of gut tube at embryonic day 13.5 (E13.5) and in nascent crypts after villus emergence happens at E16 in *p53^R172H/R172H^* embryos (Figure 2D). Taken together, these data indicate that the accumulation of mutant p53 protein in intestinal crypts is detectable throughout embryo development, and soon after birth as well as in adult mice and confirm that it is dependent on cell type and gene dosage at all ages examined.

### Expression of p53 R172H protein confined to cycling and proliferating cells of morphological normal adult small intestine

Mupt53 accumulation in crypts of the small intestine is of particular interest given the well characterised location of stem and differentiated cell populations in this dynamic tissue. This epithelium is the most rapidly self-renewing tissue in adult mammals, and can be clearly divided into three kinds of epithelium: the cycling crypt base columnar (CBC) stem cells adjacent to differentiated Paneth cells, proliferating transit-amplifying (TA) cells, and differentiated villous epithelium [20]. Our mutp53 *in situ* immunostaining indicated that mutp53 accumulated in the cycling CBC and rapid proliferating TA cells (Figure 3A, orange and brown arrows) but not in the Paneth cells and differentiated villous cells of morphological normal small intestine (Figure 3A, Cyanic and blue arrows). Stem cell marker Lgr5 is exclusively expressed in cycling columnar cells at the crypt base which is accepted as a stem cell marker of small intestine [20]. We found no change of Lgr5 mRNA expression among *p53^R172H/R172H^*, *p53^+/+^* and *p53^KO/KO^* mouse small intestine, examined by qRT-PCR and by FISH procedures. Lgr5 mRNA Expression was confined to the CBC population in all these three genotypes (Suppl Figure 3A, 3B).

To further validate mutp53 protein accumulation in stem cells of the small intestine, we isolated intestinal crypts from mice of the various *p53* genotypes and cultured them *in vitro* as organoids. We could not detect p53 by immunostaining in organoids from mice that were p53-null (data not shown). However, the organoids from *p53^R172H/R172H^* mice stained strongly for p53 while those from *p53^R172H/KO^* mice showed weak p53 immunopositivity (Figure 3B). Therefore, the organoids showed the same trend of mutant p53 accumulation as that seen in the mice.

Next we further explored whether p53 R172H affects cell proliferation in morphological normal tissues. We co-stained mutp53 and PCNA by immunostaining, more PCNA positive cells were found in crypts of p53 R172H mouse than in p53 wild type mice, although the intensity of PCNA staining in both p53 wild type and p53 R172H mutant is similar. The mutp53 positive staining cell population was always found within the PCNA expressing cell population (Figure 3C).

### p53 R172H protein accumulates rapidly after induction by ionising radiation but clears more slowly than wild type p53

Given that p53 is often stabilised upon activation [27,28], studying p53 accumulation *in vivo* could provide information on p53 induction upon drug treatment that would be relevant for the optimisation of chemotherapeutic regimens. To this end, studying the intestine is particularly important because 20-40% of all adverse effects arising from drug treatment are attributed to events in the small and large intestine [29]. For example, p53 plays a major role in the apoptosis of stem cells within the intestinal crypt [30,31], which can contribute to gastrointestinal toxicity [32]. The crypts of the small intestine also contain proliferating cells that are sensitive to ionizing radiation [31,33].

**Figure 4:**
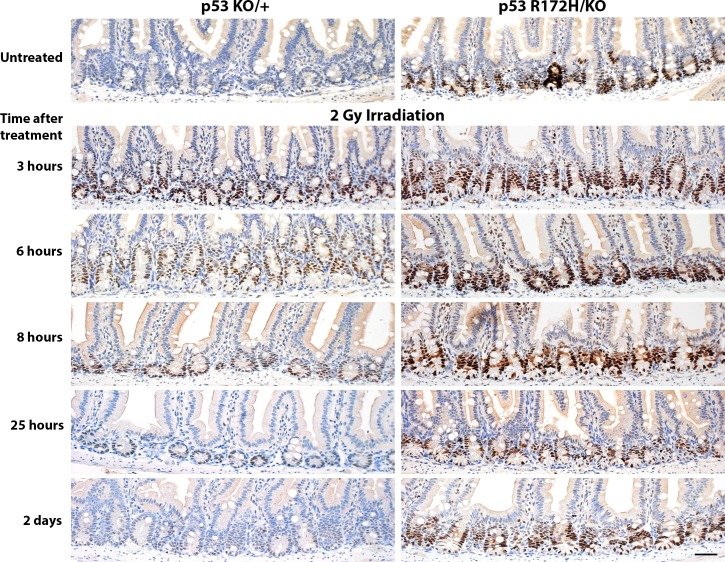
Time course of p53 protein accumulation and clearance after low-dose ionising radiation *p53^KO/+^* and *p53R^172H/KO^* mice were irradiated with 2 Gy and sacrificed at the indicated time-points. The small intestines were harvested and prepared for immunohistological analysis, then stained for p53. Scale bar: 50 μm.

Given that we observed distinct mutp53 expression in the small intestine that was dependent on gene dosage, we sought to use the small intestine of these p53 mutant mice as an experimental system to examine p53 induction. We first subjected the mice to whole body ionising radiation and performed a time course experiment to compare the response of wild type p53 protein in *p53^KO/+^* mice with that of mutant p53 in their *p53^R172H/KO^* littermates. Just 3 hours after 2 Gy ionising radiation, we could detect elevated levels of both wild type and mutant p53 protein levels in the same crypt cells in which high p53 R172H protein levels had been observed in *p53^R172H/R172H^* mice (Figure 4). Irradiation induces apoptosis and the accompanying loss of cellularity can complicate analysis of immunohistochemical data. Nonetheless, it is still clear that basal protein levels of wild type p53 were restored 8 hours after irradiation (Figure 4, left panel). In contrast, p53 R172H protein levels in most crypts took up to 25 hours to return to basal levels (Figure 4, right panel). Therefore, ionising radiation rapidly induced both wild type and mutant p53 protein in intestinal crypts, but wild type p53 protein returned to basal levels more quickly than did mutant p53.

### Nutlin induces the rapid accumulation of p53 R172H protein in intestinal crypts

**Figure 5:**
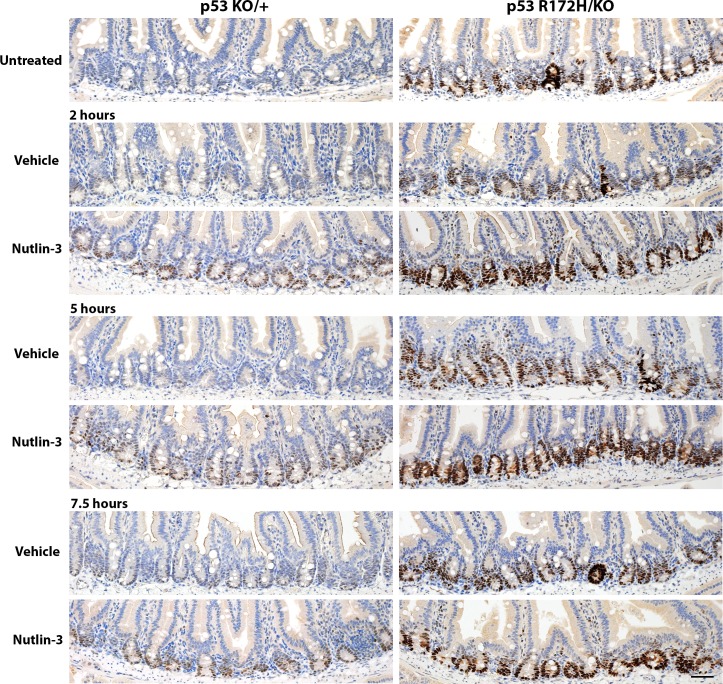
Time course of p53 protein accumulation after nutlin treatment Nutlin or vehicle control was administered to *p53^KO/+^* and *p53R^172H/KO^* mice by oral gavage. The mice were sacrificed at the time-points specified and the small intestines harvested and prepared for immunohistological analysis, then stained for p53. To facilitate comparisons, the top row reproduces the images of the nonirradiated controls in Figure 4 and in Figure 2. Scale bar: 50 μm.

We then tested whether we could similarly detect p53 induction after drug treatment. We used the small molecule nutlin, which blocks the interaction between p53 and its major negative regulator Mdm2, thereby preventing p53 degradation and inducing its activity [16]. As in the experiment with ionising radiation, we compared p53 protein levels in the small intestines of *p53^KO/+^* mice with those in their *p53^R172H/KO^* littermates at various time-points after drug administration. We could detect slight accumulation of p53 wild type protein 2 hours onward after nutlin treatment (Figure 5, left panel) whereas there was a clear increase in mutant p53 protein levels detectable 2 hours after nutlin administration and this was sustained at the 7.5 hour time-point (Figure 5, right panel). These data affirm the utility of the p53 mutant mice as a sensitive system to study p53 accumulation *in vivo*, although the p53 wild type mice are still valuable for studies of specific p53 target gene activation. They also confirm that the degradation of mutant p53 is due to the action of Mdm2 since it is inhibited by nutlin.

### Loss of Mdm2 results in even higher levels of p53 expression but p53 is still restricted to proliferating cells in the small intestine

**Figure 6:**
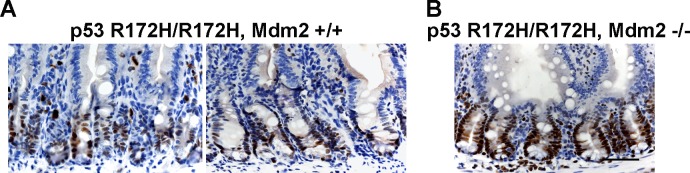
Increased mutp53 protein level in Mdm2 −/− mouse small intestine **A.** p53 R172H/R172H, Mdm2+/+ and **B.** p53R172H/R172H, Mdm−/− adult mice were sacrificed and small intestine were embedded in paraffin and sections were stained for mutp53. Scale bar: 50 μm.

In prior work on both the *p53^R172H/R172H^* mice and *p53^R172H/R172H^* zebrafish we were not able to detect mutant p53 accumulation in normal tissues though it was readily seen when these animals were crossed onto Mdm2-null backgrounds [25, 26]. We at first considered that this readily detection of mutant p53 accumulation in mice with Mdm2 could be due to the effect of other background genes of the p53 mutant mice but comparison of the staining methods used encouraged us to re-examine tissues from these mice using the sensitive antibodies and processing methods developed in the current studies. These showed that the differences in the current results are indeed due to the increased sensitivity of the current protocols. As shown in Figure 6A staining for p53 is indeed detectable in the crypt cells of the mice previously described as negative for p53. The striking increase in intensity of p53 staining in the Mdm2-null background (Figure 6B) does however completely confirm the earlier conclusions of the importance of Mdm2 in regulating mutant p53 levels *in vivo*. In support however of the additional regulation of p53 by transcriptional control we note that even in these intensely stained specimens p53 is confined to the crypt cells and is not expressed in the differentiated cells of the villi.

## DISCUSSION

**Figure 7:**
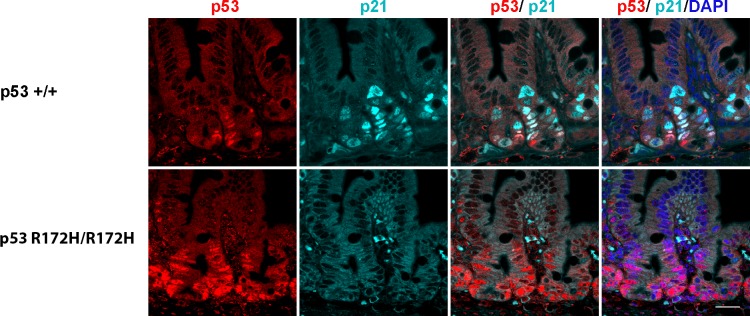
p21 protein accumulation in p53 wild type but not in p53 R172H mouse small intestines after 8 Gy irridiation Co-labeling of p53 and p21 protein in p53 wild type and p53 R172H mouse small intestines after 1 day with 8 Gy irridiation with p53 and p21 antibodies. Scale bar: 50 μm.

Previous reports have shown that the levels of mutant p53 protein are often increased in tumours but not in normal tissue, both in mice [25] and in zebrafish [34]. Our study is the first to describe elevated mutant p53 protein levels in non-cancerous mouse tissues, which may not have previously been detected due to differences in immunostaining protocols and in the p53 antibody used [24, 35]. Mutp53 protein is regulated similarly to wild type p53 in our data (Figure 4, 5) and others [25, 36]. It is induced by stress and genotoxic damage such as irradiation and degraded after ubiquitination by its major negative regulator, the ubiquitin ligase Mdm2 [25, 35]. Previous data indicated that the transcriptionally inactive mutant p53 cannot induce Mdm2 expression, which could partly explain the longer half-life of the protein in cells after induction [35]. There is no expression of p21 protein in p53 R172H mice as expected (Figure 7), indicating p53 R172H mutant mouse model we generated also lost its p53 transcriptional capacity which is consistent with previous reports [35].

Many different types of cancer show a high incidence of p53 mutations, leading to the expression of mutant p53 proteins. Although we are unable to validate whether the properties and induction of the mutp53 protein in these morphologically normal tissues are the same as that of the mutp53 protein in tumours, we did find that increased mutp53 protein accumulation is more relevant to cell and tissue types with high proliferative rate such as small intestine, thymus, spleen and anagen hair follicles. The accumulation of mutp53 protein is not spontaneous or uncontrolled, it occurs in a cell- and tissue-specific manner (Figure 1). The high accumulation of the mutp53 protein within the crypts is remarkable. We stained large numbers of sections from small intestine of different p53 R172H mice, we generally only detected elevated mutp53 protein in cycling CBC and rapidly proliferating TA cells within the small intestinal crypts (Figures 3). Our study is the first to describe elevated mutp53 protein levels in CBC and TA cells of intestinal crypts. Although we do not understand whether and how elevated mutant p53 proteins might perturb intestinal stem cells and affect tumorigenesis, this p53 R172H mouse model will provide a valuable tool for understanding the role of mutant p53 protein in the cancer initiation. In human specimens clusters of intensely stained p53 positive nuclei have been seen in clones present in skin [37] and fallopian tube (p53 signatures) [38]. Sequencing of these signatures has shown that they contain mutant p53. While high level expression of mutant p53 is associated with pre-neoplastic conditions and mutant p53 can act as a dominant transforming oncogene our studies suggest that this is not a very penetrant state. Mice expressing high levels of mutant p53 in the stem and proliferating cells of their intestine from birth do not show overt neoplastic effects in this tissue.

The mutp53 protein was not detected in differentiated Paneth cells and villous epithelium of small intestine as well as in other differentiated cells. Differentiated Sertoli cells and spermatocytes were also immuno-negative for mutp53, while spermatogonia which are undifferentiated male germ cells express elevated mutp53 protein. In stratified epithelia, such as nonglandular stomach epithelium and skin, mutp53 was detected in basal undifferentiated layers of these epithelia but not in differentiated suprabasal layers (Figure 1). This observation is consistent with previously published data in mouse embryonic stem cells that p53 protein levels and activity decrease upon differentiation [39, 40] and that it is more difficult to induce p53 activity in differentiated cells [40]. This phenomenon was mostly considered due to the post-translational regulation, which leads to down-regulation of p53 protein in differentiated cells. However in this study, whether treated by nutlin or ionising irradiated, mutp53 protein cannot be induced in differentiated villi and is always confined to the crypts of the small intestine. This is even true when the Mdm2 gene is inactivated suggesting strongly that the lack of p53 expression may be due to inhibition of the p53 promoter in differentiated cells

The anatomy of the small intestinal crypt and villus are uniquely suited to study the properties of various cell populations such as proliferating cells in crypts and differentiated cells in villi of small intestine. It still remains to be determined how p53 is down-regulated after cell differentiation. Abnormal development and incomplete differentiation are hallmarks of cancer, loss of differentiation may be linked to p53 disruption in tumourigenesis. Enforced differentiation in p53 mutant cancer may become a powerful therapeutic tool in cancer chemotherapy and drug discovery. This is because strong evidence suggests that the high level expression on mutant p53 drives the growth of the cancer and blocking its expression inhibits tumour growth [41]. Our results may also go a long way towards explaining the widely discussed variability of p53 expression in human tumours [42]. Clearly as shown here the dosage of mutant p53, the activity of Mdm2 and the activity of the p53 promoter, which is in turn linked to the state of proliferation, can along with the sensitivity of the staining protocol used have profound effects on the intensity of p53 staining observed.

## MATERIALS AND METHODS

### Mice

All mouse experiments were approved by the A*STAR Institutional Animal Care and Use Committee (IACUC) and performed in compliance with IACUC regulations. The *Trp53* knockout mice and *p53^R172H^* mutant mice were generated by crossing mice expressing Cre under the control of the β*-actin* promoter [43] with *Trp53* conditional knockout mice [44] and *p53^LSL^*•*^R172H^* conditional mutant mice [35, 45] respectively. The generation of the *p53R^172H/R172H^*; *Mdm2^−/−^* mice was reported previously [25]. *p53^R172H^* mice maintained on a mixed 129S_4_/SvJae x C57BL/6 background were used for p53 IHC staining. Genotyping was performed by PCR analysis of DNA from ear clips obtained at the time of weaning. Two-month-old (mo) mice of the appropriate genotypes were subject to total body irradiation using a single dose of 2 Gy and 8 Gy. 2 mo mice with same genotypes were treated with nutlin-3 (Nutlin, 200 mg/kg, Oral gavage). The mice were then sacrificed at the specified times. To prevent infection, mice sacrificed more than 24 hours after irradiation received 0.5 mg/ml amoxicillin, starting 3 days prior to irradiation. Mouse tissues for immunohistochemistry (IHC) and immunofluorescent staining were fixed in 10% Neutral Buffered Formalin and embedded in paraffin. Mouse tissues for fluorescence *in situ* hybridization (FISH) were fixed in cold 4% paraformaldehyde in phosphate buffered saline (PBS) and submerged overnight at 4°C in 30% sucrose/4% paraformaldehyde, embedded in OCT and stored in −80C. All tissues analysed appeared normal at the time of necropsy.

### Immunohistochemistry and microscopy imaging

Immunostaining was performed on formalin-fixed paraffin-embedded (FFPE) 5 μm sections. We used commercial rabbit anti-p53 (CM5, 1:500, Leica Biosystems, Germany) primary antibody and the p53 IHC procedure was performed by auto-staining machine (Leica Bond-max, Leica Biosystems, Germany) to ensure reproducibility of staining between experiments, the procedure was performed according to the manufacturer's instructions. The most critical step for p53 IHC staining is that we used EDTA based pH 9.0 antigen retrieval solution to exposure antigen epitopes. Home-made polyclonal rabbit anti-p53, commercial monoclonal mouse anti-p53 (1C12, 1:200, #2425S, Cell Signaling Technology, Danvers, USA), rabbit anti-PCNA (1:100, sc-7907, Santa Cruz), and mouse anti-p21 (1:20, F5, Santa Cruz) primary antibodies and anti-rabbit/mouse Alexa Fluor 568/488 (1:500, Invitrogen, California, USA) secondary antibodies were used for immunofluorescent staining. IHC Images were captured with a Zeiss AxioImager upright microscope using 20x and 40x objective lens. Data presented are representative of results obtained from at least 3 mice per group. Immunofluorescent staining images were observed using an Olympus FV1000 upright confocal microscope and captured at 405nm, 488nm, and 568nm using 40x and 100x objective lens, processing with FV10-ASW 3.0 Viewer software.

### Protein extraction and western blot

Mechanically separated crypts and villi of small intestine [46], were homogenized and lysed using RIPA buffer (25mM Tris•HCl pH 7.6, 150mM NaCl, 1% NP-40, 1% sodium deoxycholate, 0.1% SDS, Thermo Scientific, #89901) supplemented with protease inhibitor cocktail (Roche). Supernatants were collected after centrifugation at 14,000 r.p.m for 1 hour. Protein concentration was determined by the BCA method (Pierce, Thermo Scientific). Proteins were subjected to SDS−PAGE and immunoblot analysis. Blots were probed sequentially with primary and secondary antibodies at the following dilutions: anti-p53 at 1:1000 (1C12, Cell Signaling), anti-Actin at 1:5000 (Sigma-Aldrich Corporation, St. Louis, USA). Secondary HRP-conjugated anti-mouse and anti-rabbit were used at 1:10 000 (GE Healthcare, Chalfont St Giles, UK). Proteins were detected by incubation with ECL substrate (Amersham Bioscience, Piscataway, USA) for 5 min and chemiluminescence was visualized by STORM imaging system (Amersham, Pleasanton, USA).

### Total RNA isolation and qRT-PCR

Separated crypts of small intestine were homogenized and put into QIAzol (Qiagen, Hilden, Germany). Total RNA was extracted using the RNeasy Universal Plus Kit (Qiagen) according to the manufacturer's instruction. Each quantitative PCR was performed in duplicate for each primer set. Relative transcript amounts were calculated by the ΔCT method using GAPDH as a reference gene.

Primer sequences: p53 forward GTTATGTGCACGTACTCTCCTC, reverse CGTCATGTGCTGTGACTTCT. Lgr5 forward CCACAGCAACAACATCAGGT, reverse AACAAATTGGATGGGGTTGT. GAPDH forward GGAGAAACCTGCCAAGTATGA, reverse CAACCTGGTCCTCAGTGTAGC.

### FISH hybridization and microscopy imaging

The FISH procedure was performed according to previously published methods [47]. 7 μm cryo-sections were cut for hybridizations. Lgr5 mRNA Stellaris probe (mouse: NM_010195) was designed by Probe Designer at www.singlemoleculefish.com and synthesized by Biosearch Technologies. The FISH probe set consists of 96 TMR fluorophore labelled oligonucleotides. DAPI nuclear dye was included during the final wash. Images were captured at 405nm and 568nm using 100x objective len by an Olympus FV1000 upright confocal microscope.

### *In vitro* culture of organoids from intestinal crypts

Intestinal crypts were isolated and cultured *in vitro* as described previously [48]. Organoids were washed and fixed in 4% formaldehyde for an hour, then transferred to 70% ethanol before embedding in paraffin.

## SUPPLEMENTARY MATERIAL FIGURES



## References

[1] Lane DP (1992). Cancer. p53, guardian of the genome. Nature.

[2] Haupt Y, Maya R, Kazaz A (1997). Mdm2 promotes the rapid degradation of p53. Nature.

[3] Montes de Oca Luna R, Wagner DS, Lozano G (1995). Rescue of early embryonic lethality in mdm2-deficient mice by deletion of p53. Nature.

[4] Jones SN, Roe AE, Donehower LA (1995). Rescue of embryonic lethality in Mdm2-deficient mice by absence of p53. Nature.

[5] Guo L, Liew HP, Camus S (2013). Ionizing radiation induces a dramatic persistence of p53 protein accumulation and DNA damage signaling in mutant p53 zebrafish. Oncogene.

[6] Pant V, Xiong S, Jackson JG (2013). The p53-Mdm2 feedback loop protects against DNA damage by inhibiting p53 activity but is dispensable for p53 stability, development, and longevity. Genes & development.

[7] Fisher CJ, Gillett CE, Vojtesek B (1994). Problems with p53 immunohistochemical staining: the effect of fixation and variation in the methods of evaluation. Br J Cancer.

[8] Goh AM, Coffill CR, Lane DP (2011). The role of mutant p53 in human cancer. The Journal of pathology.

[9] Malkin D, Li FP, Strong LC (1990). Germ line p53 mutations in a familial syndrome of breast cancer, sarcomas, and other neoplasms. Science.

[10] Srivastava S, Zou ZQ, Pirollo K (1990). Germ-line transmission of a mutated p53 gene in a cancer-prone family with Li-Fraumeni syndrome. Nature.

[11] Martins CP, Brown-Swigart L, Evan GI (2006). Modeling the therapeutic efficacy of p53 restoration in tumors. Cell.

[12] Ventura A, Kirsch DG, McLaughlin ME (2007). Restoration of p53 function leads to tumour regression *in vivo*. Nature.

[13] Xue W, Zender L, Miething C (2007). Senescence and tumour clearance is triggered by p53 restoration in murine liver carcinomas. Nature.

[14] Issaeva N, Bozko P, Enge M (2004). Small molecule RITA binds to p53, blocks p53-HDM-2 interaction and activates p53 function in tumors. Nat Med.

[15] Shangary S, Qin D, McEachern D (2008). Temporal activation of p53 by a specific MDM2 inhibitor is selectively toxic to tumors and leads to complete tumor growth inhibition. Proc Natl Acad Sci U S A.

[16] Vassilev LT, Vu BT, Graves B (2004). *In vivo* activation of the p53 pathway by small-molecule antagonists of MDM2. Science.

[17] Lin SA, Barker N (2011). Gastrointestinal stem cells in self-renewal and cancer. J Gastroenterol.

[18] Barker N, van de Wetering M, Clevers H (2008). The intestinal stem cell. Genes Dev.

[19] Potten CS (1975). Kinetics and possible regulation of crypt cell populations under normal and stress conditions. Bull Cancer.

[20] Barker N, van Es JH, Kuipers J (2007). Identification of stem cells in small intestine and colon by marker gene Lgr5. Nature.

[21] Marshman E, Booth C, Potten CS (2002). The intestinal epithelial stem cell. Bioessays.

[22] Ireland H, Houghton C, Howard L (2005). Cellular inheritance of a Cre-activated reporter gene to determine Paneth cell longevity in the murine small intestine. Dev Dyn.

[23] Birch JM, Hartley AL, Tricker KJ (1994). Prevalence and diversity of constitutional mutations in the p53 gene among 21 Li-Fraumeni families. Cancer Res.

[24] Lang GA, Iwakuma T, Suh YA (2004). Gain of function of a p53 hot spot mutation in a mouse model of Li-Fraumeni syndrome. Cell.

[25] Terzian T, Suh YA, Iwakuma T (2008). The inherent instability of mutant p53 is alleviated by Mdm2 or p16INK4a loss. Genes & development.

[26] Chua JS, Liew HP, Guo L (2015). Tumor-specific signaling to p53 is mimicked by Mdm2 inactivation in zebrafish: insights from mdm2 and mdm4 mutant zebrafish. Oncogene.

[27] Coates PJ, Lorimore SA, Lindsay KJ (2003). Tissue-specific p53 responses to ionizing radiation and their genetic modification: the key to tissue-specific tumour susceptibility?. J Pathol.

[28] Kaeser MD, Pebernard S, Iggo RD (2004). Regulation of p53 stability and function in HCT116 colon cancer cells. J Biol Chem.

[29] Zeino Z, Sisson G, Bjarnason I (2010). Adverse effects of drugs on small intestine and colon. Best Pract Res Clin Gastroenterol.

[30] Qiu W, Carson-Walter EB, Liu H (2008). PUMA regulates intestinal progenitor cell radiosensitivity and gastrointestinal syndrome. Cell Stem Cell.

[31] Merritt AJ, Potten CS, Kemp CJ (1994). The role of p53 in spontaneous and radiation-induced apoptosis in the gastrointestinal tract of normal and p53-deficient mice. Cancer Res.

[32] Potten CS (2004). Radiation, the ideal cytotoxic agent for studying the cell biology of tissues such as the small intestine. Radiat Res.

[33] Komarova EA, Kondratov RV, Wang K (2004). Dual effect of p53 on radiation sensitivity *in vivo*: p53 promotes hematopoietic injury, but protects from gastro-intestinal syndrome in mice. Oncogene.

[34] Guo L, Liew HP, Camus S (2013). Ionizing radiation induces a dramatic persistence of p53 protein accumulation and DNA damage signaling in mutant p53 zebrafish. Oncogene.

[35] Olive KP, Tuveson DA, Ruhe ZC (2004). Mutant p53 gain of function in two mouse models of Li-Fraumeni syndrome. Cell.

[36] Suh YA, Post SM, Elizondo-Fraire AC (2011). Multiple stress signals activate mutant p53 *in vivo*. Cancer Res.

[37] Jonason AS, Kunala S, Price GJ (1996). Frequent clones of p53-mutated keratinocytes in normal human skin. Proc Natl Acad Sci U S A.

[38] Xian W, Miron A, Roh M (2010). The Li-Fraumeni syndrome (LFS): a model for the initiation of p53 signatures in the distal Fallopian tube. J Pathol.

[39] Sabapathy K, Klemm M, Jaenisch R (1997). Regulation of ES cell differentiation by functional and conformational modulation of p53. EMBO J.

[40] Menendez S, Goh AM, Camus S (2011). MDM4 downregulates p53 transcriptional activity and response to stress during differentiation. Cell Cycle.

[41] Lee MK, Teoh WW, Phang BH (2012). Cell-type, dose, and mutation-type specificity dictate mutant p53 functions *in vivo*. Cancer cell.

[42] Hall PA, Lane DP (1994). p53 in tumour pathology: can we trust immunohistochemistry?—Revisited!. J Pathol.

[43] Lewandoski M, Martin GR (1997). Cre-mediated chromosome loss in mice. Nat Genet.

[44] Jonkers J, Meuwissen R, van der Gulden H (2001). Synergistic tumor suppressor activity of BRCA2 and p53 in a conditional mouse model for breast cancer. Nature genetics.

[45] O'Gorman S, Dagenais NA, Qian M (1997). Protamine-Cre recombinase transgenes efficiently recombine target sequences in the male germ line of mice, but not in embryonic stem cells. Proc Natl Acad Sci U S A.

[46] Mariadason JM, Nicholas C, L'Italien KE (2005). Gene expression profiling of intestinal epithelial cell maturation along the crypt-villus axis. Gastroenterology.

[47] Itzkovitz S, Lyubimova A, Blat IC (2012). Single-molecule transcript counting of stem-cell markers in the mouse intestine. Nature cell biology.

[48] Sato T, Vries RG, Snippert HJ (2009). Single Lgr5 stem cells build crypt-villus structures *in vitro* without a mesenchymal niche. Nature.

