# Supplementary material for: Mutant p53 accumulates in cycling and proliferating cells in the normal tissues of p53 R172H mutant mice
Source: Oncotarget. 2015 Jul 22;6(20):17968–80. doi: 10.18632/oncotarget.4956 (PMC4627229; doi:10.18632/oncotarget.4956)
Supplement: Supplementary file 1 [file oncotarget-06-17968-s001.pdf]

Mutant p53 accumulates in cycling and proliferating cells in the normal tissues of p53 R172H mutant mice

Supplementary Material

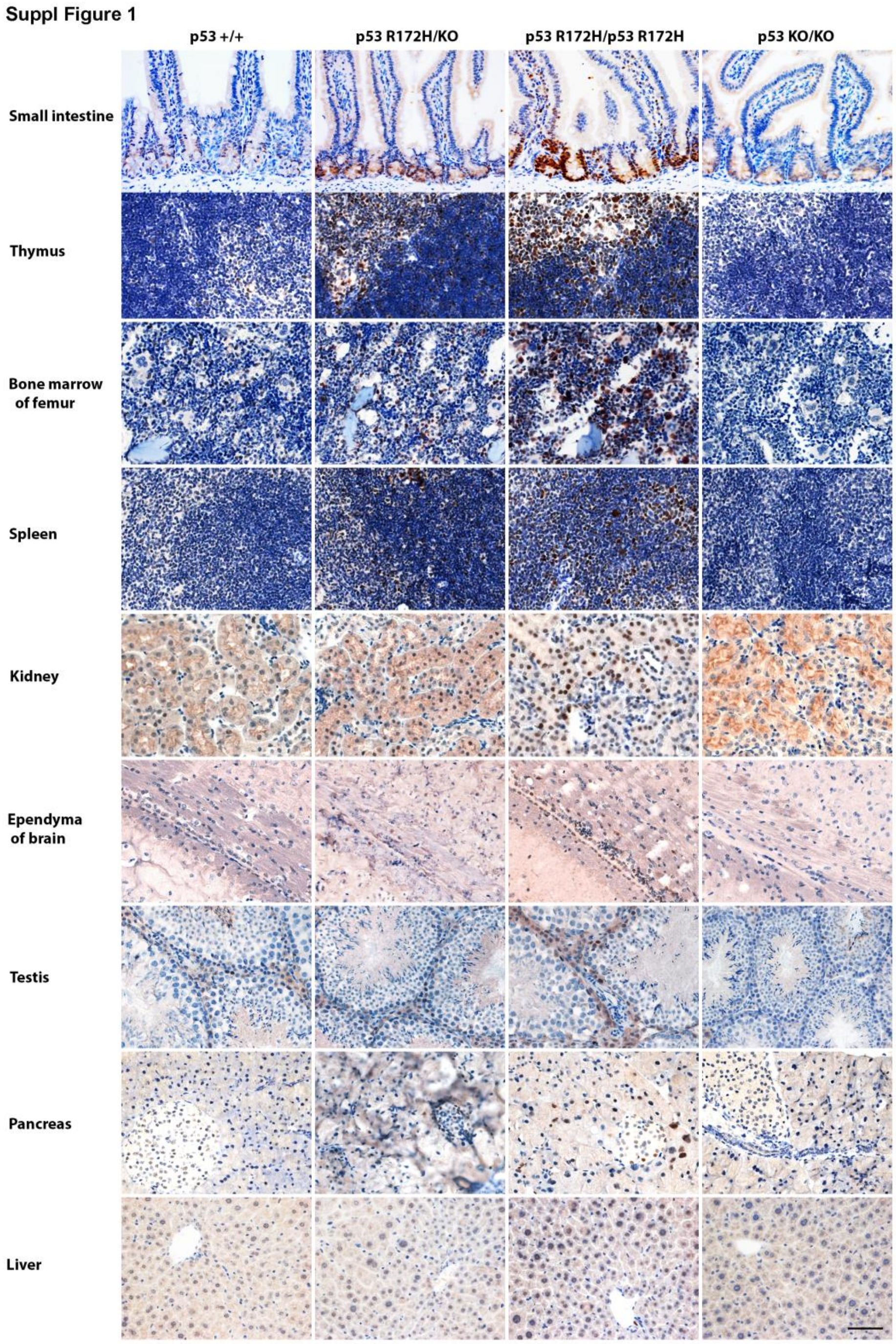

Suppl Figure 1: p53 R172H protein accumulates in multiple tissues in a manner dependent on gene dosage.

Multiple tissues were harvested from mice with the indicated genotypes. Sections were cut and stained for p53 in organs of small intestine, thymus, femur, spleen, kidney, ependyma of brain, testis, pancreas & island of Langerhans and liver. Scale bar: 50 µm.

Suppl Figure 2

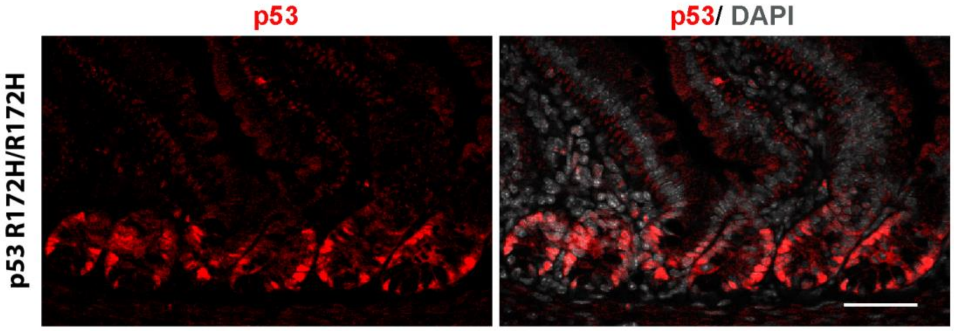

Suppl Figure 2: High p53 R172H expression in crypts of small intestine validated by immunofluorescent staining. Paraffin sections from p53 R172H/R172H mouse small intestine were stained by our home-made rabbit polyclonal anti-p53 (primary antibody) and Anti-rabbit Alexa Fluor 568 (secondary antibody), DAPI for nuclear staining. Scale bar: 50 µm.

Suppl Figure 3

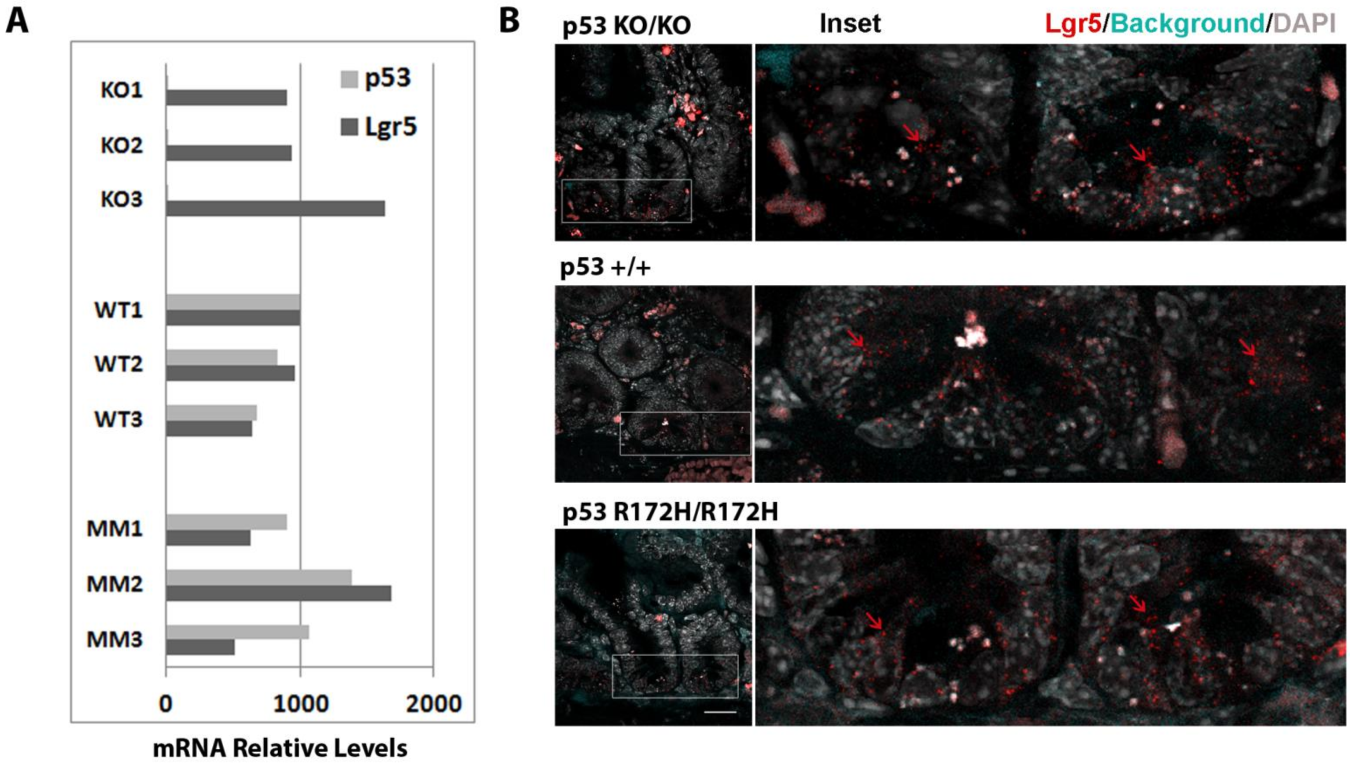

Suppl Figure 3: Lgr5 mRNA expression in small intestines of different p53 genotypes.

A. Total mRNA was extracted from crypts of small intestines in of *p53*$^{+/+}$ (WT), *p53*$^{KO/KO}$ (KO) and *p53*$^{R172H/R172H}$ (MM) mice. qRT-PCR was performed to detect relative mRNA expression levels of p53 and Lgr5 genes. **B.** Lgr5 mRNA in situ detection was performed by FISH technique in small intestines of *p53*$^{+/+}$, *p53*$^{KO/KO}$ and *p53*$^{R172H/R172H}$ mice. Scale bar: 50 µm.
